# Supplementary material for: Acceptability of a nurse-led, person-centred, anticipatory care planning intervention for older people at risk of functional decline: A qualitative study
Source: PLoS One. 2021 May 20;16(5):e0251978. doi: 10.1371/journal.pone.0251978 (PMC8136649; doi:10.1371/journal.pone.0251978)
Supplement: S1 File — (DOCX) [file pone.0251978.s001.docx]

**Additional File 1. Consolidated criteria for reporting qualitative studies (COREQ): 32-item checklist**

| Domain 1: Research team and reflexivity |  |
| --- | --- |
| Personal Characteristics |  |
| 1. Interviewer/facilitator Which author/s conducted the interview or focus group? | 1^ST^ author |
| 2. Credentials What were the researcher’s credentials? E.g. PhD, MD | PhD |
| 3. Occupation What was their occupation at the time of the study? | Research Assistant (RA) |
| 4. Gender Was the researcher male or female? | Female |
| Experience and training |  |
| 5. What experience or training did the researcher have? | Refer to TIDier checklist |
| Relationship with participants |  |
| Relationship established |  |
| 6. Was a relationship established prior to study commencement? | No. |
| Participant knowledge of the  interviewer | |
| 7. What did the participants know about the researcher? e.g. personal goals, reasons for doing the research | They knew that RA was going to talk to them about their views of, and recommendations for, the ACP intervention. |
| Interviewer characteristics |  |
| 8. What characteristics were reported about the interviewer/facilitator? e.g. Bias, assumptions, reasons and interests in the research topic | Experienced, female researcher; observed reflexivity to minimize bias |
| Domain 2: study design |  |
| Theoretical framework |  |
| Methodological orientation and Theory |  |
| 9. What methodological orientation was stated to underpin the study? e.g. grounded theory,  discourse analysis, ethnography, phenomenology, content analysis | Thematic analysis |
| Participant selection |  |
| 10. Sampling. How were participants selected? e.g. purposive, convenience, consecutive, snowball | Purposive / Snowball. |
| 11. Method of approach. How were participants approached? e.g. face-to-face, telephone, mail, email | A telephone call to arrange for T2 visit for quant. data collection and qual. interview |
| 12. Sample size. How many participants were in the study? | There were 65 participants in the full study at baseline. 34 of those were in the intervention arm, and these were interviewed about their views on the intervention. |
| 13. Non-participation. How many people refused to participate or dropped out? Reasons? | No attrition. |
| Setting |  |
| 14. Setting of data collection. Where was the data collected? e.g. home, clinic, workplace | Data was collected at participants’ homes. |
| 15. Presence of non-participants Was anyone else present besides the participants and researchers? | Other family members were present in the house, but only in one case was a spouse present during interview in the same room. |
| 16. Description of sample. What are the important characteristics of the sample? e.g. demographic data, date | Age 70+, 2+ chronic conditions, 4+ long-term medications, PRISMA frailty score of ≥3, gender, marital status, date of interviews |
| Data collection |  |
| 17. Interview guide. Were questions, prompts, guides provided by the authors? Was it pilot tested? | Yes, in the section ‘Interview Schedule’ in Methods. This study is the pilot. |
| 18. Repeat interviews Were repeat interviews carried out? If yes, how many? | No. |
| Audio/visual recording |  |
| 19. Did the research use audio or visual recording to collect the data? | Interviews were audio recorded and transcribed verbatim. |
| 20. Field notes. Were field notes made during and/or after the interview or focus group? | Yes. |
| 21. Duration. What was the duration of the interviews or focus group? | 60 minutes on average. |
| 22. Data saturation. Was data saturation discussed? | No. |
| 23. Transcripts returned. Were transcripts returned to participants for comment and/or correction? | No. |
| Domain 3: analysis and findings |  |
| Data analysis |  |
| Number of data coders |  |
| 24. How many data coders coded the data? | Two. |
| Description of the coding tree |  |
| 25. Did authors provide a description of the coding tree? | Yes. |
| Derivation of themes |  |
| 26. Were themes identified in advance or derived from the data? | Derived from the data in a bottom-up, inductive approach. |
| 27. Software. What software, if applicable, was used to manage the data? | NVivo-12. |
| 28. Participant checking. Did participants provide feedback on the findings? | No. |
| Reporting |  |
| 29. Quotations presented. Were participant quotations presented to illustrate the themes / findings? Was each  quotation identified? e.g. participant number | Yes. Participants IDs denote jurisdiction and urbanicity. |
| 30. Data and findings consistent. Was there consistency between the data presented and the findings? | Yes. |
| 31. Clarity of major themes. Were major themes clearly presented in the findings? | Yes. |
| 32 Clarity of minor themes. Is there a description of diverse cases or discussion of minor themes? | Yes. |
|  |  |

A. Tong et al.

Downloaded from https://academic.oup.com/intqhc/article/19/6/349/1791966 by guest on 14 September 2020
