# Supplementary material for: Acceptability of a nurse-led, person-centred, anticipatory care planning intervention for older people at risk of functional decline: A qualitative study
Source: PLoS One. 2021 May 20;16(5):e0251978. doi: 10.1371/journal.pone.0251978 (PMC8136649; doi:10.1371/journal.pone.0251978)
Supplement: S3 File — (DOCX) [file pone.0251978.s003.docx]

**S3. Table 2:** Participant Characteristics Details

| **Count** | **ID** | **Gender** | **Age** | **Prisma score** | **Jurisdiction** | **Intervention / Control** | **Urban / Rural** | **Attrition** |
| --- | --- | --- | --- | --- | --- | --- | --- | --- |
| **1** | MO003NI | M | 78 | 3 | NI | I | R |  |
| **2** | MO004NI | M | 87 | 4 | NI | I | R |  |
| **3** | MO005NI | F | 76 | 4 | NI | I | R |  |
| **4** | MO006NI | F | 88 | 5 | NI | I | R |  |
| **5** | MO008NI | M | 73 | 3 | NI | I | R |  |
| **6** | LB001NI | F | 83 | 5 | NI | I | U |  |
| **7** | LB002NI | M | 76 | 4 | NI | I | U |  |
| **8** | LB003NI | M | 91 | 6 | NI | I | U |  |
| **9** | LB004NI | M | 77 | 3 | NI | I | U |  |
| **10** | LB005NI | M | 80 | 3 | NI | I | U |  |
| **11** | LB007NI | F | 80 | 4 | NI | I | U |  |
| **12** | LB008NI | F | 79 | 4 | NI | I | U |  |
| **13** | LB009NI | M | 79 | 3 | NI | I | U |  |
| **14** | LB010NI | M | 78 | 3 | NI | I | U |  |
| **15** | LB011NI | F | 72 | 3 | NI | I | U |  |
| **16** | CG001NI | M | 74 | 5 | NI | C | R |  |
| **17** | CG002NI | F | 82 | 3 | NI | C | R |  |
| **18** | CG003NI | M | 90 | 6 | NI | C | R |  |
| **19** | CG004NI | M | 76 | 5 | NI | C | R |  |
| **20** | CG005NI | F | 82 | 5 | NI | C | R | T1 |
| **21** | CG006NI | F | 77 | 4 | NI | C | R |  |
| **22** | CG007NI | F | 85 | 4 | NI | C | R | T2 |
| **23** | CG008NI | M | 84 | 3 | NI | C | R |  |
| **24** | CD001NI | M | 85 | 4 | NI | C | U | T1 |
| **25** | CD002NI | F | 72 | 5 | NI | C | U |  |
| **26** | CD003NI | M | 94 | 6 | NI | C | U |  |
| **27** | CD005NI | M | 77 | 6 | NI | C | U |  |
| **28** | CD006NI | F | 87 | 4 | NI | C | U | T2 |
| **29** | CD008NI | M | 73 | 3 | NI | C | U |  |
| **30** | CD009NI | M | 83 | 6 | NI | C | U |  |
| **31** | CD010NI | F | 87 | 5 | NI | C | U |  |
| **32** | E32854ROI | M | 73 | 4 | ROI | I | U |  |
| **33** | E38659ROI | M | 78 | 3 | ROI | I | U |  |
| **34** | E39603ROI | F | 76 | 4 | ROI | I | U |  |
| **35** | E39713ROI | M | 88 | 6 | ROI | I | U |  |
| **36** | E49587ROI | M | 74 | 3 | ROI | I | U |  |
| **37** | E53448ROI | M | 80 | 6 | ROI | I | U |  |
| **38** | E54137ROI | M | 78 | 3 | ROI | I | U |  |
| **39** | E59405ROI | F | 75 | 3 | ROI | I | U |  |
| **40** | E69601ROI | M | 75 | 4 | ROI | I | U |  |
| **41** | E84283ROI | F | 72 | 3 | ROI | I | U |  |
| **42** | F34839ROI | F | 75 | 5 | ROI | I | R |  |
| **43** | F36988ROI | M | 73 | 3 | ROI | I | R |  |
| **44** | F44050NI | F | 83 | 5 | ROI | I | R |  |
| **45** | F66391ROI | F | 87 | 3 | ROI | I | R |  |
| **46** | F73221ROI | F | 88 | 6 | ROI | I | R |  |
| **47** | F75177ROI | M | 77 | 3 | ROI | I | R |  |
| **48** | F82139ROI | F | 88 | 6 | ROI | I | R |  |
| **49** | F84377ROI | F | 77 | 5 | ROI | I | R |  |
| **50** | F91087ROI | M | 83 | 5 | ROI | I | R |  |
| **51** | G60900ROI | M | 82 | 3 | ROI | C | R |  |
| **52** | G69639ROI | M | 83 | 4 | ROI | C | R |  |
| **53** | G74439ROI | M | 85 | 3 | ROI | C | R |  |
| **54** | H16761ROI | M | 79 | 3 | ROI | C | U |  |
| **55** | H18950ROI | F | 92 | 5 | ROI | C | U |  |
| **56** | H23593ROI | F | 84 | 4 | ROI | C | U |  |
| **57** | H31978ROI | F | 76 | 3 | ROI | C | U |  |
| **58** | H38952ROI | F | 79 | 5 | ROI | C | U |  |
| **59** | H47308ROI | F | 87 | 3 | ROI | C | U |  |
| **60** | H64190ROI | M | 83 | 5 | ROI | C | U | T1 |
| **61** | H68915ROI | F | 74 | 5 | ROI | C | U |  |
| **62** | H70989ROI | F | 84 | 5 | ROI | C | U |  |
| **63** | H74939ROI | F | 80 | 5 | ROI | C | U |  |
| **64** | H77603ROI | M | 84 | 3 | ROI | C | U |  |
| **65** | H86346ROI | M | 73 | 4 | ROI | C | U |  |
