# Supplementary material for: Acceptability of a nurse-led, person-centred, anticipatory care planning intervention for older people at risk of functional decline: A qualitative study
Source: PLoS One. 2021 May 20;16(5):e0251978. doi: 10.1371/journal.pone.0251978 (PMC8136649; doi:10.1371/journal.pone.0251978)
Supplement: Attachment — Submitted filename: Response to Reviewers.docx [file pone.0251978.s004.docx]

**Response to Reviewers**

The authors would like to express their sincere gratitude to the editors and reviewers for giving of their valuable time to review our submission and providing their helpful suggestions for revision, all of which we have taken on board, and endeavoured to implement and incorporate, thus making it a stronger manuscript. We have addressed each point raised during the review and responded to it with the relevant remedial action and response as indicated below.

| **Points raised by Editors and Reviewers** | **Authors’ response and action** |
| --- | --- |
| Journal requirements:   1. Please ensure that your manuscript meets PLOS ONE’s style requirements, including those for file naming. The PLOS ONE style templates can be found at   <https://journals.plos.org/plosone/s/file?id=ba62/PLOSOne_formatting_sample_title_authors_affiliations.pdf>  <https://journals.plos.org/plosone/s/file?id=wjVg/PLOSOne_formatting_sample_main_body.pdf>  <https://journals.plos.org/plosone/s/submission-guidelines> | THE AUTHORS HAVE ENDEAVOURED TO ENSURE THAT THE MANUSCRIPT MEETS PLOS ONE’S STYLE REQUIREMENTS. |
|  |  |
| In your revised cover letter, please address the following prompts:   2. We note that you have indicated that data from this study are available upon request.   PLOS only allows data to be available upon request if there are legal or ethical restrictions on sharing data publicly. For information on unacceptable data access restrictions, please see <http://journals.plos.org/plosone/s/data-availability#loc-unacceptable-data-access-restrictions>.  a) If there are ethical or legal restrictions on sharing a de-identified data set, please explain them in detail (e.g., data contain potentially identifying or sensitive patient information) and who has imposed them (e.g., an ethics committee). Please also provide contact information for a data access committee, ethics committee, or other institutional body to which data requests may be sent.  b) If there are no restrictions, please upload the minimal anonymized data set necessary to replicate your study findings as either Supporting Information files or to a stable, public repository and provide us with the relevant URLs, DOIs, or accession numbers. Please see <http://www.bmj.com/content/340/bmj.c181.long> for guidelines on how to de-identify and prepare clinical data for publication. For a list of acceptable repositories, please see <http://journals.plos.org/plosone/s/data-availability#loc-recommended-repositories>. | OUR COVER LETTER WILL NOW INDICATE THAT WE HAVE SUBMITTED OUR ANONYMISED INTERVIEW DATA AS SUPPLEMENTARY FILES. |
| 3. (Point 3 is a repeat of point 2.) |  |

**Additional Editor Comments:**

| Please to take care with formatting and proof reading and use of acronyms ROI and NI - please state respective country names in full for international readers to define acronyms when first used in line 14 … island of Ireland, northern Ireland (NI) and the Republic of Ireland (ROI). | THE AUTHORS HAVE FOLLOWED THE PLOS FORMATTING GUIDELINES, AND HAVE CAREFULLY PROOF-READ THE MANUSCRIPT.  THE ABSTRACT NOW DISPLAYS COUNTRY NAMES IN FULL, FOLLOWED BY THEIR ACRONYMS AT FIRST MENTION IN LINES 21 AND 22:  THE FEASIBILITY CRCT INVOLVED EIGHT GENERAL PRACTITIONER (GP) PRACTICES AS CLUSTER SITES, STRATIFIED BY JURISDICTION, FOUR IN NORTHERN IRELAND (NI) (TWO INTERVENTION, TWO CONTROL), AND FOUR IN THE REPUBLIC OF IRELAND (ROI) (TWO INTERVENTION, TWO CONTROL). ‘ISLAND OF IRELAND’ DOES NOT USE AN ACRONYM. |
| --- | --- |
| **Please improve the clarity of the reporting in the methods.** | THE AUTHORS HAVE ENDEAVOURED TO IMPROVE THE CLARITY OF THE REPORTING IN THE METHOD SECTION. |
| The methods state paper follows COREQ guidelines. Please include as a supplementary file your completed COREQ checklist for your paper and indicate in the manuscript (see supplementary file 1). | THE COMPLETED COREQ CHECKLIST IS NOW INCLUDED AS A SUPPLEMENTARY FILE (S3) AND THIS IS INDICATED IN TEXT (PAGE 5). |
| Please give detail on the intervention - the only detail given is the number of visits, that conducted by a research nurse who assessed health domains and liaised with GP and pharmacist to devise an anticipatory care plan. This is too brief for a complex intervention. Please review to the TIDier checklist for intervention description and replication. | MORE DETAIL HAS NOW BEEN PROVIDED ON THE INTERVENTION IN SECTION 2.2 IN THE METHODS, IN KEEPING WITH THE TIDieR Checklist, WHICH HAS BEEN COMPLETED AND IS ALSO PROVIDED AS A SUPPLEMENTARY FILE (S4), INDICATED ON PAGE 5. |
| How was the research nurse trained: | RESEARCH NURSES COMPLETED A THREE-DAY TRAINING PROGRAMME DESIGNED TO ORIENT THEM TO THE INTERVENTION AND STUDY PROCEDURES. THE TRAINING WAS FACILITATED BY A CLINICIAN EXPERT IN THE FIELD. THIS IS NOW STATED ON PAGE 6. |
| Was the intervention manualized for consistency, did this incorporate any evidence-based interventions to e.g. medicine optimization i.e. STOPPfrail. If your population was older people with frailty, were the frailty syndromes considered in the assessment e.g., continence, falls, reduced mobility. Or a general 'health assessment' - what informed the components. if this is detailed in the published trial protocol, please give sufficient detail of the intervention for the reader to understand what it comprised. | YES, THE INTERVENTION WAS MANUALISED WHICH IS REFLECTED IN THE TRAINING OF THE NURSES IN TERMS OF STANDARDISED ASSESSMENT, REPORTING, AND ENGAGEMENT WITH GP. THE MEDICATION REVIEW WAS BASED ON BEST EVIDENCE. THE ACP ASSESSMENT WAS CONDUCTED USING THE STANDARDISED EASY-CARE TOOL (Philip KE, Alizad V, Oates A, Donki DB, Pitsillides C, Syddal SP, et al. Development of an EASY-Care, for brief standardized assessment of the health and care need of older people; with latest information about cross-national acceptability. J Am Med Dir Assoc. 2014;15: 42-6) WHICH INVOLVES SEVERAL DOMAINS TO ENSURE A PERSONALISED HOLISTIC APPROACH. THIS WAS SUPPLEMENTED WITH A MEDICATION REVIEW. PAGE 6 NOW PROVIDES THIS INFORMATION. |
| How was this assessment 'person-centred' - this is the main message in the conclusion, yet no detail is given as to how this was person-centered. We need understanding on the detail in the intervention that person-centred and how e.g. asked what are the person's priorities, used an evidence-based tool e.g. person-centred comprehensive PCOM i.e. ESAS/IPOS, or Staying Well Check tool <https://www.england.nhs.uk/wp-content/uploads/2017/11/dg-case-study-staying-well-check-tool.pdf> | PAGES 5-6 NOW INCLUDE: THE RESEARCH NURSES (5) WERE TRAINED BY AN EXPERT CLINICIAN. TO ENSURE A PERSONALIZED CARE APPROACH, REGISTERED NURSES FROM BOTH JURISDICTIONS COMPLETED A THREE-DAY TRAINING PROGRAMME INCLUDING STUDY OVERVIEW, PRINCIPLES AND PRACTICE OF PERSONALIZED CARE, SHARED DECISION MAKING, CONDUCTION OF A HOLISTIC ASSESSMENT WITH THE EASY-CARE TOOL, AND COMPLETING A MEDICATION REVIEW IN COLLABORATION WITH A CLINICAL PHARMACIST. |
| How was intervention person-centred: | THE STUDY AIMS, TRAINING PROVIDED TO THE RESEARCH NURSES, AND THE EASY-CARE ASSESSMENT TOOL ALL ALIGNED TO REFLECT PERSON-CENTRED CARE. THIS IS NOW BETTER EXPLAINED IN THE INTERVENTION SECTION, PAGES 5-7. |
| Did the same research nurse deliver the intervention and conduct the qualitative interviews? Please can you clarify in your methods. This is important for the rigour of your qualitative study. | THIS HAS NOW BEEN CHANGED. PAGE 5 STATES THAT THE INTERVIEWS WERE CONDUCTED BY AN EXPERIENCED RESEARCHER. PAGES 5-6 STATE THAT THE INTERVENTION WAS CONDUCTED BY SPECIALLY TRAINED RESEARCH NURSES. |
| Please state in your methods when the qualitative interviews were undertaken in relation to the feasibility trial, e.g. after completion of the intervention at XX weeks post randomisation. | Section 2.1 PAGE 5 STATES THAT THE QUALITATIVE INTERVIEWS WITH PARTICIPANTS TOOK PART AT 10-WEEK FOLLOW-UP (AUGUST TO OCTOBER 2019) IMMEDIATELY AFTER COMPLETION OF THE INTERVENTION AND AT THE SAME TIME AS TIME 2 QUANTITATIVE DATA COLLECTION. |
| Please also state if the interviews were conducted in a separate data collection time point or with the quant data. This is can be brief if detailed in the protocol. But this is important to understand the quality of your embedded qualitative study in the feasibility trial. | WE HAVE NOW INDICATED THAT THE QUALITATIVE INTERVIEW TOOK PLACE AT THE SAME TIME AS TIME 2 QUANTATIVE DATA COLLECTION. SECTION 2.1 PAGE 5. |
| PLease remove reporting on randomisation and sampling from the intervention detail. Randomisation and sampling could move to your sampling section. | THIS HAS BEEN MOVED TO THE SAMPLE SECTION. |
| In the methods 2.3 sample section - please move reporting on your sample to the results, and detail your method of sampling in the methods. | THIS HAS BEEN DONE. |
| The sampling states eligibility criteria for the trial. How were the participants for individual interviews purposively selected. Please state the criteria used. | ALL INTERVENTION PATIENTS WERE INTERVIEWED; WE HAVE NOW OMITTED THE WORD ‘PURPOSIVELY’. (PAGE 7). |
| **Section 3 Findings** Please begin with the detail reporting the participants moving this detail from the methods section. | THIS HAS BEEN DONE. |
| Please provide more detail on your sample - mean/SD or median and IQR for PRISMA with interpretation e.g. moderate frailty; mean/median age and respective measures of dispersion, ethnicity; and illness factors e.g. main diagnosis group by ICD-10 chapter headings i.e. cardiovascular disease. or if multiple conditions state to give some sense of why considered this patient group likely to benefit from ACP. This data is important to understand your sample and applicability of your findings for other populations. | IN LINE WITH UK NATIONAL HEALTH SERVICE RECOMMENDATIONS THE BEST PRACTICE PRISMA-7 SCREENING TOOL WAS APPLIED TO IDENTIFY PATIENTS AT RISK OF FUNCTIONAL DECLINE / FRAILTY. PRISMA-7 IDENTIFIES THOSE WITH A SCORE OF THREE OR HIGHER AS AT INCREASED RISK FOR FRAILTY. THIS HAS NOW BEEN EXPLAINED IN THE ‘SAMPLE’ SECTION. MEANS AND SD FOR PRISMA SCORES AND FOR AGE HAVE NOW BEEN INCLUDED IN THE FINDINGS SECTION, AS HAVE GENDER AND ETHNICITY. AS PER INCLUSION CRITERIA ALL PARTICIPANTS HAD TWO OR MORE CHRONIC CONDITIONS.  THE SUITABILITY OF, AND RATIONALE FOR, THE CHOSEN PATIENT GROUP IS PROVIDED IN THE INTRODUCTION. |
| I would suggest putting this demographic data in Table 1: Participant characteristics. | THIS TABLE (TABLE 1: PARTICIPANT CHARACTERISTICS) IS NOW INCLUDED (PAGE 7). |
| Also please detail how many interviews was a caregiver present. | ONE CARE PARTNER ONLY WAS PRESENT AT INTERVIEW. THIS HAS NOW BEEN STATED IN THE FINDINGS. |
| And please state how representative is your qualitative sample from the feasibility trial sample. How many people approached for the qualitative interview agreed to participate, what were the reasons for decline. | ALL INTERVENTION PARTICIPANTS WERE INVITED AND AGREED TO TAKE PART IN THE INTERVIEWS. THIS IS NOW STATED IN THE FINDINGS TOO. |
| Your qual work indicates that participants considered themselves 'too well' for the ACP, and those who were frailer indicated greater benefit. Please considered if the patients who agreed for interview were generally 'well' and more able to participate in this aspect of the study causing potential sampling bias. | ALL INTERVENTION PARTICIPANTS (n=34) AGREED TO PARTICIPATE IN THE QUALITATIVE INTERVIEW THUS AVOIDING SAMPLING BIAS. THIS IS NOW STATED IN THE ‘SAMPLE’ SECTION. |
| **Discussion** This is a qualitative study. Please use language describing your results aligned with qualitative methodology. For example line 496 - very effective, is quant positivist language. please use nouns/adjectives to report qual findings e.g. essential, crucial. | WE HAVE ENDEAVOURED TO REMOVE SPECIFICALLY QUANTITATIVE LANGUAGE. |
| Please review the discussion and structure to tighten the reporting on the main messages made. Key points seems the 'holistic' assessment undertaken by the research nurse and sense of being 'heard and valued', multidisciplinary team working particularly intervention of the pharmacist for medicine review/optimization. The role of the GP from your findings would seem more than 'appreciated' (line 580). Important to give a sense of how the intervention worked from the findings. | WE HAVE REVIEWED AND STRUCTURED THE DISCUSSION, FOCUSING ON THE MAIN POINTS FROM THE FINDINGS.  GP ANCHORAGE HAS INDEED BEEN IDENTIFIED AS ESSENTIAL RATHER THAN ‘APPRECIATED’. THIS HAS BEEN AMENDED ACCORDINGLY. |
| The RE-AIM framework is reported in the first para of the methods, but there is no further reference as to how RE_AIM was applied with the study e.g. used to inform design of the topic guide, data analysis, data interpreting? | RE-AIM, MORE SPECIFICALLY THE ‘ADOPTION’ COMPONENT (WE HAVE CLARIFIED THAT NOW IN ‘DESIGN AND PROCEDURES’), WERE USED AS A FRAMEWORK TO GUIDE THE EVALUATION, AND AS SUCH GUIDED THE TOPIC GUIDE (AS STATED IN THE SECTION ‘INTERVIEW SCHEDULE’ OF THE METHODS), AND DATA ANALYSIS (STATED IN THE ‘DESIGN AND PROCEDURE’ SECTION OF THE METHODS). |
| If RE-AIM not used in this aspect of the study please remove from the methods, or detail in your methods how applied and pick this up in the discussion. For example - How does RE_AIM relate to your 4FMA framework identified in your data analysis? | THIS HAS NOW BEEN EXPANDED UPON IN SECTION 2.4, AND TAKEN UP IN THE DISCUSSION. |
| **Conclusions** - these need to be tightened to reflect your findings. | WE HAVE TIGHTENED THE CONCLUSIONS TO CLEARLY REFLECT OUR FINDINGS. |
| **Reviewer #1:**  Thank you for the opportunity to review this manuscript. It is a very interesting topic and good to see implementation acceptability being reported for ACP. Please find following my general, and more specific comments which I feel will make this a stronger manuscript. | THE AUTHORS THANK REVIEWER #1 FOR TAKING THE TIME TO REVIEW THE MANUSCRIPT AND FOR THE VALUABLE INPUT AND RECOMMENDATIONS WHICH WE HAVE ENDEAVOURED TO FULLY INTEGRATE AND IMPLEMENT. |
| **Overall:** **Background**: You explain the background in great depth and there are some very relevant points. However, there are some statements which do not appear strictly relevant to building your argument. Reducing some of these peripheral statements would make this section stronger and improve readability | WE HAVE REMOVED STATEMENTS WHICH MAY BE CONSIDERED PERIPHERAL AND THUS NOT STRICTLY RELEVANT. |
| **Quotes**: Throughout you change formatting between quotes indented and quotes within text. It is off-putting for the reader. Decide which and stick with it. Very short quotes (a few words) within the text are fine. | QUOTES ARE NOW FREESTANDING THROUGHOUT THE FINDINGS SECTION WITH THE EXCEPTION OF VERY SHORT QUOTES. |
| **Discussion**: This feels quite long and would benefit from greater contextualization in the wider literature. | WE HAVE TIGHTENED AND CONTEXTUALISED THE DISCUSSION. |
| **Implications**: Again this feels long. The manuscript would benefit from the repetitions being cut and this section focusing on potential clinical and research impacts. You also mention health education here for the first time. Please introduce this in the Discussion and strengthen your argument with external literature. Overall you mention education five times, including in the conclusion, but this is not then mentioned in your abstract conclusions. If this is a significant implication it should be noted in the abstract. | WE HAVE REMOVED REPETITIONS AND FOCUSED ON POTENTIAL CLINICAL AND RESEARCH IMPACTS.  WE HAVE INTRODUCED HEALTH EDUCATION IN THE DISCUSSION AND BROUGHT IN WIDER LITERATURE. PATIENT EDUCATION HAS ALSO NOW BEEN INCLUDED IN THE ABSTRACT. |
| **Conclusions**: This could be more clearly linked to your key messages. | WE HAVE LINKED THE CONCLUSION MORE CLEARLY TO THE KEY MESSAGES. |
| **Formatting**: As PLOS ONE does not copyedit accepted manuscripts please do check over the full manuscript and figure for typographical errors such as use of semicolons and capitalisation etc. | WE HAVE ENDEAVOURED TO IDENTIFY AND REMOVE ALL TYPOGRAPHICAL ERRORS. |
| **Specific**: Line 10: I would have liked to see the main aim reflected here. | THE MAIN AIM OF THIS PAPER IS TO REPORT ON PATIENT ACCEPTABILITY OF THE PRIMAY CARE BASED ACP INTERVENTION. THIS IS NOW MORE CLEARLY REFLECTED HERE. |
| Lines 17-18: This is rather confusing. Is that four practices in the control and four intervention? Or four patients within each of the eight practices to control and four patients within each of the eight practices to intervention? Neither adds up to the n=34 you interviewed. I make 8 practices and 8 participants at each to be a total of 64, half of which, the intervention group, would be 32. Obviously you know how many people you interviewed so I must have misunderstood something in the way the numbers are explained. This needs clarifying throughout the manuscript. | WE HAVE NOW CLARIFIED NUMBERS OF PRACTICES AND PARTICIPANTS BOTH IN THE ABSTRACT AND IN THE METHODS SECTION. |
| Line 25-26: I do not understand the relevance of the hyphens here or later in the manuscript (e.g. Line 111) | THE HYPHENS HAVE BEEN REMOVED. |
| Line 26-28: These are not articulated the same as the main headings in your findings section (Lines 161-163). Please correct. | THIS HAS NOW BEEN CORRECTED. |
| Line 98: Can you add that participants gave informed consent here? I appreciate you say it below, but not to see it here raises a red flag to the reader. | THIS HAS BEEN ADDED. |
| Lines 105-106: See Lines 17-18 comment above. | IN LINE WITH THE REQUIREMENTS OF THIS REVIEW THIS INFORMATION HAS NOW BEEN MOVED TO THE ‘SAMPLE’ SECTION, HOWEVER, IT HAS BEEN AMENDED IN ORDER TO CLARIFY NUMBERS, AND TO BRING IT IN LINE WITH THE AMENDED ABSTRACT. |
| Line 109: Move the bracketed element after 'for inclusion'. Also not sure you need the brackets, just a separate sentence. It would make this clearer that there is more data if the reader wishes. | THE BRACKETS HAVE BEEN REPLACED BY A SEPARATE SENTENCE. |
| Line 110: How many met the inclusion criteria? Why did you decide on only 8 from each practice? What is your rationale? | 73 PATIENTS MET THE INCLUSION CRITERIA. DETAILS ON RATIONALE REGARDING SAMPLING HAVE NOW BEEN PROVIDED. |
| Line 112: What was the dose? Did some only receive one? Why didn't everyone receive the same dose? Specifying would help the reader decide the weight of the findings. | THIS DETAIL IS NOW INCLUDED IN SECTION 2.2: THE INTERVENTION, IN THE METHOD SECTION. NUMBER OF HOME VISITS DEPENDED ON COMPLEXITY OF NEEDS. |
| Line 115: Specify this was the nurse. 'They' can be confusing and imply it was the team of GP, pharmacist and nurse. If it was the team, this needs to be clarified throughout as previously it has implied it was the nurse that constructed the plan. | ‘THEY’ HAS NOW BEEN REPLACED WITH ‘THE NURSES’ HERE. |
| Line 121: Why face to face? | THIS WAS AN ERROR AND SHOULD HAVE READ “… WERE SELECTED TO TAKE PART IN A FACE-TO-FACE INTERVIEW.” WE HAVE NOW REMOVED THE EXPRESSION ALTOGETHER. |
| Line 131: Can you reference some of this literature? | WE HAVE NOW RECTIFIED THE OMISSION AND INCLUDED REFERENCES FOR THE RE-AIM FRAMEWORK. |
| Line 132: Did you use PPI? If not, it may be useful to explain your justification for this. | YES, PPI WAS USED. THIS HAS NOW BEEN INCLUDED HERE. |
| Line 144: When? Also January 2019? | YES, POST ETHICAL APPROVAL AND PRIOR TO INDIVIDUAL BASELINE DATA COLLECTION. BASELINE WAS STAGGERED OVER SEVERAL MONTHS BETWEEN FEBRUARY AND JUNE 2019. |
| Line 148: Who conducted the data analysis. All members of the research team? Is the research team the writing team? | THE FIRST AUTHOR IN COLLABORATION WITH ANOTHER MEMBER OF THE RESEARCH TEAM (PAGE 11). THE INTERVENTION NURSES ARE NOT PART OF THE WRITING TEAM. |
| Line 165: Make it clearer that this is a conceptual framework which you have developed and give more details. Also, potentially mention as an output in your abstract. | WE HAVE NOW CLARIFIED THAT THIS IS A NEW CONCEPTUAL FRAMEWORK WHICH WE HAVE DEVELOPED AND PROVIDED A LITTLE MORE DETAIL. WE HAVE ALSO INCLUDED THE FRAMEWORK IN THE ABSTRACT. |
| Line 172-176: Describing what is in the chart doesn't add anything. Try and give a high level summary for this theme here. | WE HAVE EDITED THIS PARAGRAPH TO REFLECT THE SUGGESTED CHANGES. |
| Line 194: Where is the evidence for this? | EVIDENCED THROUGH PARTICIPANT FEEDBACK DURING INTERVIEWS, AND ILLUSTRATED THROUGH THE QUOTES THROUGHOUT THE FINDINGS SECTION. THIS HAS NOW BEEN STATED IN TEXT. |
| Line 226: Impossible is a very strong word. Challenging? Difficult? | THIS HAS BEEN CHANGED TO ‘CHALLENGING’. |
| Line 268-271: I find this statement very uncomfortable. How did you analyse negative and positive mindsets? I appreciate participants gave what appeared to be two different views. Maybe report on that rather than your interpretation of what mindset that meant they had. | THIS SECTION HAS NOW BEEN RE-WRITTEN IN LINE WITH THE REVIEWER’S SUGGESTION. |
| Line 277: This is the first mention of frailty. Are you diagnosing frailty as a syndrome, if so, how, or saying some patients were more susceptible to functional decline? I would change this title or quantify. Perhaps Impact of (fluctuating?) mental and physical capacity? | WE HAVE CHANGED THE TITLE OF THIS SECTION AS PER YOUR SUGGESTION. |
| Line 282: Was capacity one of your inclusion criteria? If not did that impact the intervention and your study? You do not mention this in your limitations or elsewhere. | CAPACITY WAS NOT AN INCLUSION CRITERION PER SE BUT THE ABILITY TO COMPLETE AN ENGLISH LANGUAGE POSTAL QUESTIONNAIRE. THIS IS NOW INCLUDED IN THE SAMPLE SECTION. |
| Line 283-284: This sentence is confusing to me. (The following section makes perfect sense.) I am unsure what this togetherness of physical and mental capacity means, or where your evidence is to clarify it. | WE HAVE REMOVED THIS SENTENCE. |
| Line 324: The use of the word subsequent makes the sentence somewhat confusing. | WE HAVE REMOVED THE WORD ‘SUBSEQUENTLY’. |
| Line 337 and 505: Again, please clarify what you mean by the term frailty in this context. | THE WORD FRAIL HAS BEEN REPLACED WITH ‘INFORM’ IN THESE SENTENCES [WHAT WAS MEANT WITH ‘FRAIL COHORT’ WAS THAT PARTICIPANTS IN THIS STUDY HAD A PRISMA-7 FRAILTY SCORE OF 3+.] |
| Line 357: Is spouse the right word when your example is regarding adult children? | ‘SPOUSE’ WAS NOW REPLACED WITH ‘FAMILY MEMBER’. |
| Line 366-368: Can you evidence this with a quote? | A SUPPORTING QUOTE HAS NOW BEEN INCLUDED HERE. |
| **Reviewer #2:** I think this is a useful study to examine the effects of nurse-led Anticipatory care planning. I did not understand the following points, so I would like to ask for additional explanation. | THE AUTHORS THANK REVIEWER #2 FOR TAKING THE TIME TO REVIEW THE MANUSCRIPT AND FOR THE VALUABLE INPUT AND RECOMMENDATIONS WHICH WE HAVE ENDEAVOURED TO FULLY INTEGRATE AND IMPLEMENT. WE HAVE RESPONDED TO THE REQUEST FOR ADDITIONAL EXPLANATIONS BELOW. |
| In the Intervention part, it is stated that the GP was divided into the intervention group and the control group by Feasibility RCT, but if this study is a cluster RCT design, please describe the flow chart of the participants. If it's not a cluster RCT design, shouldn't this description be included in the sample? | PARTICIPANT RECRUITMENT HAS NOW BEEN DETAILED IN THE SAMPLE SECTION. |
| In addition, although it is divided into an intervention group and a control group, please describe the correspondence to the control group. | WE HAVE NOW INCLUDED THIS DETAIL IN THE SAMPLE SECTION: PARTICIPANTS WERE CONTACTED BY A MEMBER OF THE RESEARCH TEAM TO INFORM THEM OF THEIR ALLOCATION TO THE CONTROL VERSUS THE INTERVENTION GROUP AFTER CONSENT HAD BEEN OBTAINED AND BASELINE STANDARDISED INTERVIEW COMPLETED. |
| If this study is cluster RCT design, I think the sample size is small. | THIS IS THE RECOMMENDED SAMPLE SIZE FOR FEASIBILITY PURPOSES AND IS DETAILED IN OUR PROTOCOL. |
| Please specify the meaning and effect of collecting data separately for the intervention group and the control group. | THIS PAPER REPORTS ON THE PATIENT ACCEPTABILITY OF THE ACP INTERVENTION. TO ELICIT THIS, INTERVENTION PARTICIPANTS WERE INTERVIEWED REGARDING THEIR VIEWS AND PERCEPTIONS OF THE INTERVENTION. NO SUCH QUALITATIVE DATA WERE COLLECTED FROM THE CONTROL GROUP WHO DID NOT RECEIVE THE INTERVENTION.  QUANTITATIVE DATA WAS COLLECTED SIMULTANEOUSLY FROM INTERVENTION AND CONTROL PARTICIPANTS, HOWEVER, THIS WILL BE THE TOPIC OF ANOTHER PAPER. |
| [NOTE: If reviewer comments were submitted as an attachment file, they will be attached to this email and accessible via the submission site. Please log into your account, locate the manuscript record, and check for the action link "View Attachments". If this link does not appear, there are no attachment files.]  While revising your submission, please upload your figure files to the Preflight Analysis and Conversion Engine (PACE) digital diagnostic tool, <https://pacev2.apexcovantage.com/>. PACE helps ensure that figures meet PLOS requirements. To use PACE, you must first register as a user. Registration is free. Then, login and navigate to the UPLOAD tab, where you will find detailed instructions on how to use the tool. If you encounter any issues or have any questions when using PACE, please email PLOS at [figures@plos.org](mailto:figures@plos.org). Please note that Supporting Information files do not need this step. | N/A  WE HAVE UPLOADED FIG 1 TO PACE AND APPENDED THE RESULTING DOCUMENT WITH OUR RESUBMISSION. |
