# Supplementary material for: Acceptability of a nurse-led, person-centred, anticipatory care planning intervention for older people at risk of functional decline: A qualitative study
Source: PLoS One. 2021 May 20;16(5):e0251978. doi: 10.1371/journal.pone.0251978 (PMC8136649; doi:10.1371/journal.pone.0251978)
Supplement: Attachment — Submitted filename: Response to Reviewers.docx [file pone.0251978.s005.docx]

**Response to Reviewers**	19/03/2021

Once again, the authors would like to express their sincere gratitude to the editors and reviewers for giving of their valuable time to review our resubmission and providing further helpful suggestions for revision, all of which we have endeavoured to take on board, implement and incorporate, thus making it a stronger manuscript. We have addressed each point raised during the second review and responded to it with the relevant remedial action and reply as indicated in the table below.

| **Points raised by Editors and Reviewers** | **Authors’ response and action** |
| --- | --- |
| **Journal Requirements:**  Please review your reference list to ensure that it is complete and correct. If you have cited papers that have been retracted, please include the rationale for doing so in the manuscript text, or remove these references and replace them with relevant current references. Any changes to the reference list should be mentioned in the rebuttal letter that accompanies your revised manuscript. If you need to cite a retracted article, indicate the article’s retracted status in the References list and also include a citation and full reference for the retraction notice. | WE HAVE REVIEWED THE REFERENCES FOR COMPLETION AND CORRECTNESS.  WE HAVE ADDED TWO MORE REFERENCES (36 AND 50) AND ADJUSTED NUMBERING THROUGHOUT. |
| **Additional Editor Comments** (if provided): | THE AUTHORS THANK THE EDITOR FOR REVIEWING THE AMENDED MANUSCRIPT AND SUGGESTIONS TO FURTHER STRENGTHEN IT. WE HAVE ENDEAVOURED TO RESPOND TO EACH POINT AS BELOW. |
| Please use language of frail rather than infirm throughout. (see #Reviewer 1 point below) | ‘INFIRM’ WAS REPLACED WITH ‘FRAIL’ THROUGHOUT (2 REPLACEMENTS MADE). |
| **Methods** Detail on the RCT needs to be in the design and procedure section including reference for the published protocol. This then details the design at the beginning of the methods for the reader to understand the intervention and qualitative interviews are part of a trial. | DETAILS ABOUT THE INTERVENTION, INCLUDING REFERENCE TO THE PUBLISHED PROTOCOL HAVE NOW BEEN MOVED TO THE BEGINNING OF THE ‘DESIGN AND PROCEDURE’ SECTION IN THE METHODS. THE PARAGRAPH ABOUT THE QUALITATIVE INTERVIEWS IMMEDIATELY FOLLOWS THIS. |
| Reporting follows COREQ guidance. This needs to be detailed in the design section as relates to the whole study reporting, not only the intervention. Can you move up from the intervention detail and report in the design subsection. | THE SENTENCE: ‘THIS PAPER REPORTS ON PATIENT ACCEPTABILITY OF THE INTERVENTION, AND FOLLOWS THE COREQ GUIDELINES FOR REPORTING QUALITATIVE RESEARCH [27] (SEE S3) AND THE TIDIER CHECKLIST [28] (SEE S4)’ HAS NOW BEEN MOVED TO SECTION 2.1 ‘DESIGN AND PROCEDURE’. |
| **Sample** Can you revise this for clarity – moving information on the trial design to the study design section. State clearly at the beginning sample eligibility, then give the detail. Such as .. | THE SENTENCE PERTAINING TO TRIAL DESIGN HAS BEEN MOVED TO THE STUDY DESIGN SECTION. THE SENTENCE DENOTING INCLUSION CRITERIA HAS BEEN MOVED TO LEAD ON FROM DESCRIPTION OF ELIGIBLITY AND INCLUSION (LINES 136-139). |
| The qualitative interviews included all participants allocated to the intervention group in the feasibility cluster RCT. Detailing on the sampling method for the trial can remain in this sub-section. | THIS INFORMATION REMAINED IN THIS SUB-SECTION. |
| Table 1 – can you include this detailed table as a supplementary file, and cite in the manuscript as supplementary. It is important information, but is not main results. Include  in the results  table 1 reporting characteristics of the qual sample (see below). | THIS TABLE IS NOW INCLUDED AS SUPPLEMENTARY FILE 5 (S5): TABLE 2. PARTICIPANT CHARACTERISTICS, AND IS REFERRED TO AS SUCH IN THE MANUSCRIPT. |
| Line 156 – please remove pre-COVID, not needed as implicit from the dates given that data collection occurred before the 2020 pandemic | THE PHRASE ‘PRE-COVID’ HAS BEEN REMOVED HERE. |
| Interview schedule - line 164 - please state how PPI supported the study and give an example to illustrate contribution to the interview schedule. Please detail if a PPI group - number, how worked with them referring to GRIPP reporting for PPI. | THE FOLLOWING SENTENCES HAVE NOW BEEN INSERTED HERE:  FOLLOWING GRIPP GUIDELINES ON PPI REPORTING [36] WE ENGAGED THREE PPI (ONE IN ROI, TWO IN NI) IN AN ADVISORY CAPACITY TO ATTEND REGULAR PROJECT TEAM MEETINGS AND TO DISCUSS PROTOCOL, ANALYSIS, AND DISSEMINATION, AS WELL AS PROGRESSION AND NEXT STEPS, SPECIFICALLY TO CONSULT ON STUDY DOCUMENTS, INCLUDING QUALITATIVE AND QUANTITATIVE INTERVIEW SCHEDULES, TO ENSURE INCORPORATION OF THE VITAL LAY PERSON PERSPECTIVE. AN EXAMPLE OF PPI INPUT TO THE QUALITATIVE INTERVIEW SCHEDULE IS THE CHANGE FROM ‘DID YOU FEEL ACTIVELY INVOLVED IN YOUR DISCUSSIONS WITH THE NURSE TO IDENTIFY YOUR HEALTHCARE NEEDS?’ TO ‘DID YOU HAVE ENOUGH INPUT IN IDENTIFYING YOUR HEALTH NEEDS AND DEVELOPING YOUR CARE PLAN?’ |
| **Findings** Can you present in the results a table 1 summary table of the qualitive interview participant characteristics e.g. Age median (range IQR), gender – proportions, average PRISMA score i.e.d median and spread, jurisdiction – proportions, allocated group etc. You can then reduce the narrative reporting on the participant results to main points and refer to the table 1. | PREVIOUS TABLE 1 WITH DETAILED PARTICIPANT DETAILS HAS NOW BEEN INCLUDED AS A SUPPLEMENTARY FILE TABLE 2 (S5) AS REQUESTED ABOVE. WE HAVE NOW INCLUDED A SUMMARY TABLE WITHIN THE TEXT (TABLE 1), SHOWING AGE MEAN AND SD, GENDER, PRISMA SCORE MEAN AND SD, AND DISTRIBUTIONS OF ROI/NI, INTERVENTION/ CONTROL, AND URBAN/ RURAL. |
| Line 210 – please remove detail on methods of data analysis. This is repeating detail given in the methods. Please report your findings to indicated what found – past tense. Not we sought to elicit – future tense as you are reporting findings.  Reporting on the themes and framework begin as a new para, Line 209 new para to report the themes identified. State suggestion below, then give the detail re the themes and the framework Our findings on the acceptability of the intervention formed five main themes:  Use of the word correct – review 1, suggested use appropriate timing. I agree with this – from your findings this is about the appropriate time for the person. Not a correct time or un-correct time which implies a biomedical perspective of disease stage. This is about the priorities for the person, not their disease stage. | DETAIL ON METHODS OF ANALYSIS HAVE BEEN REMOVED.  WE HAVE INSERTED A PARAGRAPH AT LINE 222 AND STARTED SENTENCE IN PAST TENSE (‘ANALYSIS OF INTERVIEW DATA RESULTED IN FIVE MAIN THEMES …’), OMITTING THE PREVIOUS ‘WE SOUGHT TO ELICIT’.  THE WORD ‘CORRECT’ HAS BEEN REPLACED BY ‘APPROPRIATE’ IN RELATION TO TIMING THROUGHOUT. |
| **Discussion** Limitations – line 611 – no cross jurisdictional differences, but the sample all identified as ethnically White. Transferability is limited to ethnically White. Please indicate in the limitations this point sample ethnically White. This limits transferability to Black and Minority ethnic groups. | IN LIMITATIONS, LINE 638 NOW READS: ‘AS THE SAMPLE WAS ENTIRELY ETHNICALLY WHITE TRANSFERABILITY TO OTHER ETHNIC GROUPS MAY BE LIMITED.’ |
| **Reviewer #1:**  Thank you for the opportunity to review the amended manuscript. It is, I feel, far clearer and stronger. I do have some additional comments which I believe will strengthen the manuscript further: | THE AUTHORS THANK REVIEWER #1 FOR REVIEWING THE AMENDED MANUSCRIPT AND SUGGESTIONS TO FURTHER STRENGTHEN IT. WE HAVE ENDEAVOURED TO RESPOND TO EACH POINT AS BELOW. |
| Throughout - I assume comments from the author team have been answered to everyone's satisfaction? e.g. lines 709 & 713 on the annotated copy. | APOLOGIES; IT IS UNCLEAR WHAT THIS POINT IS REFERRING TO? LINES 709 AND 713 OF THE MANUSCRIPT ARE IN THE REFERENCE SECTION. WE ARE HAPPY TO ADDRESS THIS ONCE CLARIFIED. |
| Throughout - Changing frail to infirm and changing the heading. This is perhaps a misunderstanding of my comment. I was concerned you had categorized frailty without explaining what that meant to you within the paper. You have now explained PRISMA-7 and its role in flagging frailty well and so may I ask that you go back to using the term frail, or older people living with frailty, or frail elders. Infirm has its own negative connotations. | WE HAVE CHANGED THE TERM ‘INFIRM’ BACK TO ‘FRAIL’ THROUGHOUT THE MANUSCRIPT.  THE HEADING HAS NOT CONTAINED EITHER OF THESE TERMS BUT REFLECTS THE WORDING OF THE STUDY TITLE WITH THE TERM ‘AT RISK OF FUNCTIONAL DECLINE’ WHICH IS USED THROUGHOUT THE MANUSCRIPT AS WELL. |
| Throughout - Appropriate rather than 'correct' timing. | THIS HAS BEEN AMENDED THROUGHOUT THE MANUSCRIPT (2 INCIDENTS). |
| Line 103 - The reader needs to know a bit more contextualisation. "at the same time as collecting time 2 quant data" isn't enough. | THIS SENTENCE (STARTING AT LINE 121) READS NOW: THIS INVOLVED QUALITATIVE INTERVIEWS WITH PARTICIPANTS IN THEIR OWN HOMES AT 10-WEEK FOLLOW-UP (AUGUST TO OCTOBER 2019) FOLLOWING COMPLETION OF THE INTERVENTION, DURING THE SAME VISIT AT WHICH TIME 2 QUANTITATIVE DATA WERE COLLECTED. PARTICIPANTS COMPLETED THE QUANTITATIVE QUESTIONNAIRE WITH THE RESEARCHER AND WERE THEN INTERVIEWED. |
| Lines 160 - 174 - level of detail is much better, but perhaps the actual questions could sit in a separate table/box so the reader doesn't get lost now you've added the aim of the questions. Also, may need to explain why you are using advance CP and anticipatory CP here. | THE QUESTIONS HAVE NOW BEEN PLACED IN A BOX WITHIN THE TEXT.  WE HAVE REPLACED THE MENTION OF ‘ADVANCE CARE PLANNING’ WITH ‘ANTICIPATORY CARE PLANNING’. |
| Line 163-164 - could you mention briefly how the PPI supported this? | THE FOLLOWING SENTENCE HAS NOW BEEN INSERTED HERE (STARTING AT LINE 166):  FOLLOWING GRIPP GUIDELINES ON PPI REPORTING [36] WE ENGAGED THREE PPI IN AN ADVISORY CAPACITY TO ATTEND REGULAR PROJECT TEAM MEETINGS AND TO DISCUSS PROGRESSION, NEXT STEPS, AND CONSULT ON STUDY DOCUMENTS, INCLUDING QUALITATIVE AND QUANTITATIVE INTERVIEW SCHEDULES, TO ENSURE INCORPORATION OF THE VITAL LAY PERSON PERSPECTIVE. AN EXAMPLE OF PPI INPUT TO THE QUALITATIVE INTERVIEW SCHEDULE IS THE CHANGE FROM ‘DID YOU FEEL ACTIVELY INVOLVED IN YOUR DISCUSSIONS WITH THE NURSE TO IDENTIFY YOUR HEALTHCARE NEEDS?’ TO ‘DID YOU HAVE ENOUGH INPUT IN IDENTIFYING YOUR HEALTH NEEDS AND DEVELOPING YOUR CARE PLAN?’ |
| Line 187 - much clearer. Can you add the initials of the writing team member here? | THE INITIALS OF THE TEAM MEMBER – KB – HAVE NOW BEEN INSERTED HERE. |
| Line 589 - 590 - Could you expand a little on patient education and appropriate timing, and link this to the wider literature? | LINES 601-604 NOW READ: HEALTH EDUCATION FOR OLDER ADULTS CAN BE VERY EFFECTIVE, BOTH IN TERMS OF IMPROVING INTERVENTION ADHERENCE AND POTENTIALLY IN REDUCING MORBIDITY AND EXCESS MORTALITY [40,50]. IT COULD HELP IMPROVE HEALTH LITERACY [39,42] AND ENSURE KNOWLEDGE AND UNDERSTANDING OF ACP, THUS FACILITATING TIMELY UPTAKE. |
| **Reviewer #2:**  Thank you for revise your paper. I think it's a very useful paper. Because you had revision it carefully, I was able to understand. I am waiting for a research paper with other data to be published. | THE AUTHORS THANK REVIEWER #2 FOR THEIR REVIEW AND COMMENDATION.  WE CURRENTLY HAVE ANOTHER QUALITATIVE PAPER UNDER REVIEW, AND ONE IN PREPARATION. WE ARE ALSO PREPARING A PAPER REPORTING ON ALL QUANTITATIVE ASPECTS OF THE STUDY, INCLUDING A HEALTH ECONOMIC EVALUATION. |
| **AUTHORS’ CHANGE**  **ACKNOWLEDGEMENTS** | WE HAVE ALTERED THE SENTENCE RECOGNISING THE NICRN CONTRIBUTION TO BE A MORE SPECIFIC ACKNOWLEDGEMENT WHICH NOW READS:  ‘WE THANK THE NORTHERN IRELAND CLINICAL RESEARCH NETWORK [PRIMARY CARE] (NICRN PC) FOR THE RECRUITMENT OF GP PRACTICES AND THE DELIVERY OF THE ACP INTERVENTION IN NORTHERN IRELAND.’ |
